# Quantitative measures of human *Cytomegalovirus* infection and their associations with tuberculosis disease progression and *Mycobacterium tuberculosis* infection

**DOI:** 10.1098/rstb.2024.0410

**Published:** 2025-11-06

**Authors:** Lisa Stockdale, Vivian Tamietti Martins, Gershim Asiki, Basil Sambou, Muhamed Sissoko, Uzochukwu Egere, Abdou Sillah, Beate Kampmann, Helen Fletcher, Robert Newton, Robin Basu Roy

**Affiliations:** ^1^ Oxford Vaccine Group, Department of Paediatrics, University of Oxford, Oxford, England OX3 7LE, UK; ^2^ Jenner Institute, Nuffield Department of Medicine, University of Oxford, Oxford, England OX3 7DQ, UK; ^3^ Federal University of Minas Gerais, Belo Horizonte, Minas Gerais, Brazil; ^4^ Medical Research Council/Uganda Virus Research Institute and London School of Hygiene and Tropical Medicine Uganda Research Unit, Uganda Virus Research Institute, Entebbe, Central Region, Uganda; ^5^ Vaccines and Immunity Theme, MRC Unit The Gambia, London School of Hygiene and Tropical Medicine, Fajara, Gambia; ^6^ Department of International Public Health, Liverpool School of Tropical Medicine, Liverpool, England L3 5QA, UK; ^7^ Division of Infectious Diseases and Tropical Medicine, Medical Centre of the University of Munich (LMU), Munich 80802, Germany; ^8^ German Centre for Infection Research (DZIF), Partner Site Munich, Munich 80802, Germany; ^9^ Clinical Research Department, Faculty of Infectious and Tropical Diseases, London School of Hygiene and Tropical Medicine, London WC1E 7HT, UK; ^10^ Institute for International Health, Charité - Universitätsmedizin Berlin, Germany, Berlin 10178, Germany; ^11^ Faculty of Infectious and Tropical Diseases, London School of Hygiene and Tropical Medicine, London, England WC1E 7HT, UK; ^12^ Department of Health Sciences, University of York, York, England YO10 5DD, UK; ^13^ Centre for Genomics and Child Health, Queen Mary University of London Blizard Institute, London, England E1 2AT, UK

**Keywords:** tuberculosis, cytomegalovirus, case–control, herpes virus, Uganda, The Gambia

## Abstract

Interactions between human cytomegalovirus (HCMV) and the host immune response to *Mycobacterium tuberculosis* (*M.tb*) may influence the risk of tuberculosis (TB) disease progression. Data on an association between HCMV and risk of initial infection with *M.tb* are lacking. In this correlation analysis, serological measures of HCMV were investigated in two cohorts: a TB case–control study nested within a Ugandan general population epidemiology cohort with serum samples from 25 TB disease cases up to 10 years prior to diagnosis, and a paediatric *M.tb* infection study with 22 matched pairs of highly-TB-exposed children from The Gambia, where one of each pair was infected with *M.tb* and the other was not (as determined by a tubeculin skin test (TST)). Among individuals in the Ugandan case–control study, we found a relationship between odds of progression to active TB disease and (i) increased levels of HCMV IgM (odds ratio (OR) 2.5 (99% CI 0.84–7.54) for medium tertile, and OR 3.55 (1.27–9.96) for high tertile), (ii) HCMV IgG avidity (OR 2.82 (0.88–9.01) for medium and OR 3.08 (1.25–11.82) for high), and (iii) C-reactive protein (CRP) levels (OR 1.70 (0.58–5.00) for medium and OR 3.59 (1.20–10.74) for high tertile of response). Among Gambian children, no association was found between TB infection and measures of HCMV exposure. Further evaluation of such associations in larger prospective studies and experimental testing for a causal relationship are needed.

This article is part of the discussion meeting issue ‘The indirect effects of cytomegalovirus infection: mechanisms and consequences’.

## 1. Introduction

It is estimated that one quarter of the global population is infected with *Mycobacterium tuberculosis* (*M.tb)* [1] and yet the majority of people with asymptomatic *M.tb* infection do not go on to develop active pulmonary disease. Although there are recognized risk factors for the development of tuberculosis (TB) disease (including young age [2], human immunodeficiency virus (HIV) infection [3], diabetes [4], IFN-γ deficiencies [5] and malnutrition [6]), we still do not understand why some people develop TB disease after *M.tb* infection and some do not. Infection with *M.tb* through inhalation of aerosolized bacilli is detected through a tuberculin skin test (TST), which measures skin swelling at the site of injection of *M.tb* purified protein derivative (PPD), or an interferon gamma release assay (IGRA), whereby T cells are stimulated ex vivo with *M.tb* antigens. A positive result from either one of these tests, but the absence of clinical symptoms alongside a normal chest X-ray, indicates that the individual has *M.tb* infection. For *M.tb* infection, it is generally accepted that risk is determined by the force of infection in the environment and increases with age, as cumulative exposure to the pathogen increases. Risk factors associated with progression to TB disease are incompletely understood; however, epidemiologic studies show clear age-related associations, with infants and children below the age of 5 years and adolescents being particularly at risk of developing TB disease [2]. As an intracellular pathogen, the importance of *M.tb*-specific antibodies has historically been thought to be minimal. However, evidence from a systems serology approach found that antibody-dependent function differed between latent and active TB disease [7] and this is a field of active investigation.

The influence of viral co-infections on the immune system and on risk of TB is well recognized. Concomitant HIV infection is associated with a 20.6 times increased risk of TB [8]. We and others have shown that human cytomegalovirus (HCMV) is implicated in increased risk of TB disease. Previous work by our group in the same Ugandan cohort studied here found that when over 300 samples from age- and sex-matched individuals were split into tertiles of HCMV IgG, individuals with medium levels of HCMV IgG were 2.8 times, and those with high levels of HCMV IgG 3.4 times more likely to develop TB disease in a 10 year period (*p* = 0.055 and *p* = 0.007, respectively [9]). Importantly, increased serologically determined exposure to other herpes viruses (herpes simplex virus (HSV1/2) and Epstein–Barr virus (EBV)) were not associated with any increased risk of TB disease in the same cohort [9]. Separately, an HCMV-associated IFN-γ response and CD8 T-cell activation were associated with increased TB disease risk in both infants and adolescents in South Africa [10]. In a large (*n* = 963) birth cohort in Cape Town, South Africa, acquisition of HCMV infection before the age of 12 months was associated with increased risk of TB disease after 1 year of age, and risk of TB disease was consistently greater in those with high HCMV viral loads than in those with low HCMV viral loads that were acquired before age 3 months [11]. These studies use a heterogeneous mixture of assays to determine HCMV seropositivity, ranging from HCMV-specific IgG serology, CD4 and CD8 T-cell positivity to HCMV peptides by IFN-γ release and presence of virus as measured by PCR.

There are less published data on potential associations between HCMV and TB infection. The finding that risk of *M.tb* infection in a BCG-vaccinated South African infant cohort was associated with serum markers of inflammation and immune activation commonly associated with HCMV infection did not translate into direct association with HCMV infection [12]. Despite some disappointing results from a Phase 2b HCMV vaccine efficacy trial [13], the robust HCMV vaccine development landscape [14], including an mRNA candidate [15], means that it may be possible to investigate the impact of an HCMV vaccine upon progression to TB disease in at-risk populations.

The mechanism by which HCMV infection—also known as human herpesvirus-5 (HHV-5)—might increase the risk of TB disease progression, or initial infection, is not known; however, once an individual is infected with the herpes virus, HCMV establishes lifelong latency in a variety of cell types, including those infected by *M.tb*. Despite its ubiquity (over 95% seropositive by the age 5 years in Uganda [16] and 42% by 12 months in South Africa [11]) and its association with immune variation [17], natural killer (NK) cell responses [18], T-cell activation [19], immune senescence [20] and memory inflation [21], acquired HCMV infection is usually considered benign in immunocompetent hosts. The ways in which HCMV and TB subvert the host immune response, and how these mechanisms might overlap, are explored in Olbricht *et al.* [22].

We therefore hypothesize that humoral responses to CMV infection are associated with increased risk of acquisition of TB infection and disease. Here, we further explore the relationship between HCMV serology and risk of TB disease in a Ugandan cohort, and test for associations between HCMV, mycobacteria-specific antibody-dependent NK-cell activation and *M.tb* infection in a case–control study of highly TB-exposed pairs of Gambian children with discordant infection status.

## 2. Methods

### (a) Ugandan cohort

**Figure 1.**
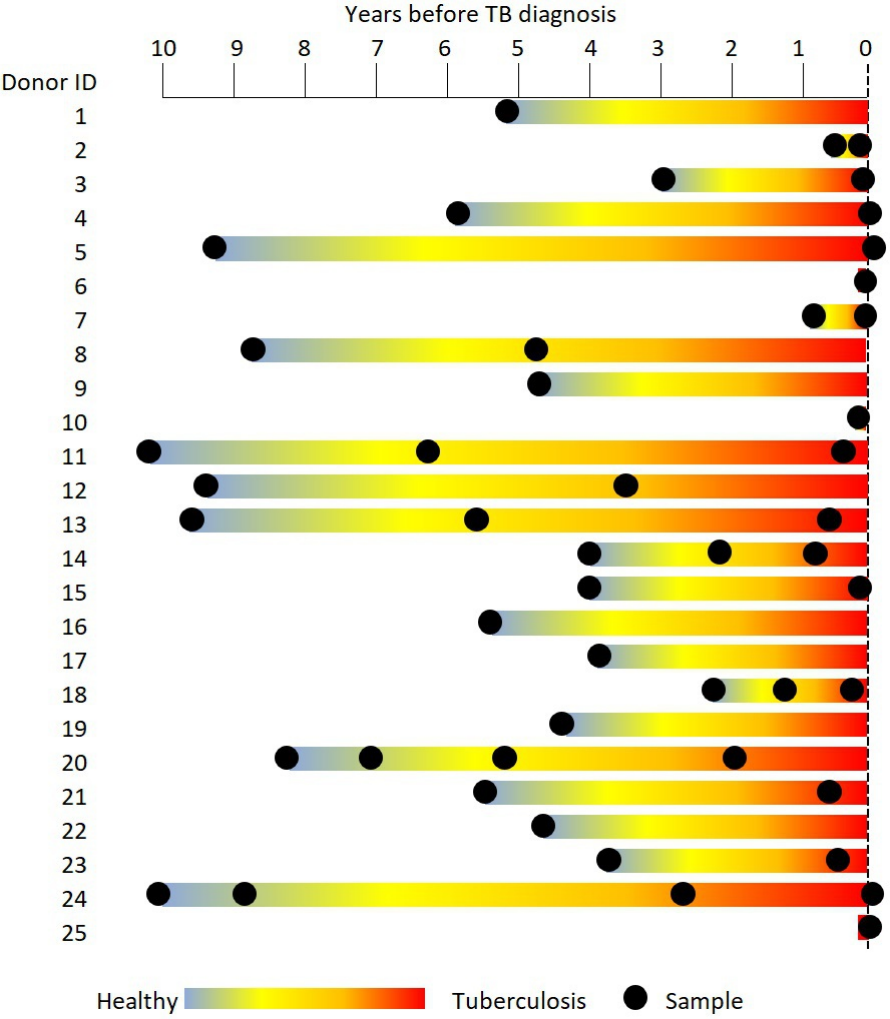
Ugandan cohort. Twenty-five individuals diagnosed with active TB disease had between one and four samples taken prior to, and at the point of TB diagnosis (total case samples *n* = 49). Black markers represent a sample.

The stored serum samples from the Ugandan cohort used for this study are described in Stockdale *et al.* [9]. The general population cohort (GPC) is a population-based open cohort established in rural Uganda in 1989 to examine trends in prevalence and incidence of HIV infections and their determinants (described by Asiki *et al.* [23]). TB disease was diagnosed through passive case identification of symptomatic individuals presenting for care at GPC clinics. The 25 individuals included here were diagnosed with sputum-positive active TB disease between 1999 and 2014. A total of 49 samples were collected from these 25 TB cases (between one and four stored serum samples per TB case) from between 10 years before and up to three months after TB diagnosis. Data were collected through annual census and blood samples were taken sporadically, as directed by specific research questions. Samples were stored at −80°C in a biobank located in Entebbe, Uganda. Figure 1 shows the timing of samples obtained from TB cases aligned with time of TB diagnosis, and table 1 shows the age, sex, HIV status and numbers of samples per individual used in this analysis. Controls who had no record of TB disease by the end of the date range studied here (2014) were selected from GPC stored serum samples collected in 2011, and were matched on known predictors of HCMV level; age, sex and HIV status at the time when sample was taken. Control samples were designated the same time before TB diagnosis as the TB case to which they were matched. Between three and six control individuals were matched per TB case sample (a maximum of one sample per control individual). Because of the ubiquity of HCMV infection within this population [16], HCMV seronegative samples were excluded (*n* = 9, all nine were non-TB control individuals with a mean age of 37 years (26.9−50.8 years); two of these nine were HIV positive). The 291 HCMV-positive samples (242 controls and 49 TB samples, grouped into 49 case–control matched sets) were included in the analyses (table 1), such that TB case individuals with multiple samples could be included in more than one set. The exposure of interest was TB disease as a binary measure.

**Table 1.** Age, sex, HIV status and numbers of samples per individual for control individuals (no TB) and case individuals (with TB disease) from the Ugandan case–control cohort. HIV, human immunodeficiency virus.

	control individuals (no TB; *n* = 242)	TB case individuals (*n* = 25)	total participants (*n* = 267)
age; mean (range), years	34.2 (2.75−56.50)	35.0 (13.10−54.90)	34.6 (2.75−56.50)
female sex; number (%)	155 (64)	15 (60)	170 (58)
HIV-infected; number (%)	53 (22)	8 (32)	61 (23)
samples per individual; mean number (range)	1 (1)	1.96 (1−4)	1.09 (1−4)
samples; total number	242	49	291

### (b) Gambian cohort

As described in [24], pairs of asymptomatic children (5–15 years old) with discordant TST status despite sleeping in the same building and with equal proximity to the same adult TB index case with smear-positive pulmonary TB were recruited in the MRC Unit, The Gambia at London School of Hygiene and Tropical Medicine (LSHTM) in 2015 (highly TB-exposed–uninfected [HEU] and highly TB-exposed–infected children [HEI]). Children (<15 years) within a cluster of a newly identified adult (>15 years) smear-positive TB index case were consented for screening of TB symptoms and TST evaluation. To ensure clear phenotypic distinction, a TST of ≥10 mm induration was considered positive and a TST <5 mm negative. Children with a TST result of 5−10mm were ineligible for inclusion in the study. Children with a positive TST were referred for further investigation, including chest X-ray, clinical review and an in-house IGRA. All participants were screened for TB symptoms at three-monthly intervals for 1 year. Highly TB-exposed–uninfected children were defined as TST negative (≤5 mm) both at study initiation and at a second TST at least three months after initial screening. There were no children with severe clinical malnutrition or immunosuppressant medication and all highly TB-exposed–infected children had a negative HIV result. Table 2 shows age and sex of matched TB infected and uninfected children included in this analysis based upon availability of serum. Gambia is a low HIV incidence country and none of the adult TB index cases in the study had HIV. The TST administration by skilled TB field workers, repeated TST in those with a result <5 mm and sustained absence of symptoms further reduced the risk of false negative TST results owing to other causes [25].

**Table 2.** Age and sex for Gambian matched TB-infected and -uninfected children.

characteristic	highly TB-exposed–uninfected children	highly TB-exposed–infected children
*n*	25	25
sleeping proximity to index case		
same room	1	1
same house	24	24
mean age years (range)	8.7 (5−14)	10 (6−14)
females number (%)	12 (48)	11 (44)

### (c) Ethical approval and consent to participate

Ethical approval for the Ugandan study was obtained from the London School of Hygiene and Tropical Medicine (refs 10 000 and 10 643), the Uganda Virus Research Institute Research and Ethics Committee (ref. GC/127/15/06/512) and from the Uganda Council for Science and Technology. Written consent for the use of clinical records and biological samples for research purposes was obtained from all GPC participants following the Uganda National Council of Science and Technology guidelines.

Ethical approval for the Gambian study was obtained from The Gambia Government/MRC Joint Ethics Committee (SCC1405 and SCC1273) and the Imperial College Healthcare Tissue Bank (R13071).

### (d) Cytomegalovirus-specific IgG, IgM and IgG avidity

Measurement of HCMV IgG, IgM and IgG avidity was executed using commercial kits (IgG (RE57061) and IgM (RE57071): IBL, Germany; and IgG avidity (EI 2570-9601-1-G): EuroImmune, Germany) and optical density (OD) readings were measured using a BioTek ELx808. The resulting measurement (in units (U) for HCMV IgG and IgM, and relative avidity index (RAI; %) for HCMV IgG avidity) was calculated based on a standard curve from the calibration sera and based on kit cut-offs. For HCMV IgG and IgM, seropositivity was determined by measures above 11U and seronegative samples were determined as having levels below 9U. Between 9 and 11U, samples were deemed equivocal and required re-testing, with any falling into the 9−11U measure being classed as seronegative.

### (e) Total IgG

Total immunoglobulin was measured in the Ugandan samples only by ELISA. Serum samples were diluted 1 : 8 × 105 and samples plus IgG standards (134.4−8.4 ng ml^−1^) were incubated on plates coated with mouse anti-human IgG (Abcam ab200699). After washing and incubating with goat anti-human Fc (Abcam ab97225), plates were read using a BioTek ELx808. OD values were converted into grams per litre by using the standard curve on each plate.

### (f) C-reactive protein

C-reactive protein (CRP) was measured using a commercial ELISA kit (R&D Systems, USA; DCRP00). OD readings were measured using a BioTek ELx808 and data were calculated in mg l^−1^.

### (g) Serum cytokines

The Luminex multiplex cytokine platform (R&D Systems, USA) was used to determine the concentrations of IP-10, IL-10, IFN-γ, IL-4, IL-13 and IL-12 in serum samples. Bio-Plex manager software v. 6.1 was used for bead acquisition and analysis of median fluorescence intensity (MFI). MFI was converted to pg ml^−1^ using the software.

### (h) Antibody-dependent natural killer cell activation

To assess antigen-specific antibody-dependent natural killer cell activation (ADNKA), 96-well Nunc maxisorp ELISA plates (Thermo Fisher) were coated overnight at 4°C with PPD (WHO 1st international Standard for PurifiedProtein Derivative (PPD) of *M. tuberculosis*; PPD-T, NIBSC; see https://nibsc.org/documents/ifu/PPDT.pdf) or ESAT-CFP10 fusion protein (gift from Professor Tom Ottenhof, Leiden University Medical Centre, The Netherlands; manufactured in-house) at a concentration of 2.0 and 2.5 µg ml^−1^, respectively, in carbonate/bicarbonate buffer.

Plates were washed three times with phosphate-buffered saline (PBS) and blocked with 5% BSA in PBS for 2 h at 37°C. Undiluted serum samples were plated in duplicate. Following 2 h incubation at 37°C, the plates were washed again and 1 x105 Natural Killer NK-92® cell line retroviral-transduced to express human CD16. PTA-8836 American Type Culture Collection, as described in [26], were added in the presence of Brefeldin A (10 µg ml–1, Sigma-Aldrich, Golgi Stop (BD Biosciences)) and CD107a (1 : 20 dilution; PE, clone H4A3, BD Biosciences). A sample of cells was separately stained with CD56 (1 : 1000 dilution; BV786, clone NCAM16, BD Biosciences) and CD16 (1 : 10 dilution; AF594, clone GRM-1, Santa Cruz Biotechnology) to verify consistent expression of CD16. After 5 h of incubation, cells were transferred to V-bottom plates and stained for FACS analysis. Live NK cells were identified by fixable LIVE/DEAD staining (1 : 500 dilution; R780, BD Biosciences). Cells were fixed and data acquired using a BD Fortessa. Percentages of CD107a + NK cells relative to control wells with PPD or ESAT6/CFP10 fusion protein and blocking buffer were only determined using FlowJo software (v. 10.7.1, BD Life Sciences). Controls, plated in triplicate, consisted of a pool of four highly TB-exposed, TB-infected Gambian children (positive control), a pool of five non-BCG-vaccinated UK children aged between 6 months and 2 years from the Oxford Vaccine Group Biobank (Ref OVCB/053), and blank (DMEM Media only (Thermo Fisher, D6546)).

### (i) Statistical analyses

For the Ugandan cohort, analyses were conducted on all 291 samples to investigate differences between individuals who progressed to diagnosis with active TB disease and those who remained TB free. Associations between herpesvirus IgM, IgG avidity, CRP and TB disease were investigated using a conditional logistic regression model conditioned on the 49 matched case–control sets. For tertile analyses, ranges of measurements among the measured samples were split into three groups, with equal numbers of measurements in each group. To account for multiple comparisons, 99% confidence intervals (CIs) are reported and a *p* value of 0.01 is considered to represent strong evidence to reject the null hypothesis. A robust standard error was used in conditional regression analysis to account for the fact that some TB case individuals contributed more than one sample.

For the Gambian cohort, following a non-significant Wilcoxon matched pairs signed rank test to test for differences in age between the matched highly exposed–uninfected (HEU) and highly exposed–infected (HEI), a univariate analysis was conducted to investigate differences in median measurements between matched HEU and HEI children using a Wilcoxon matched pairs signed rank test.

All analyses were performed using either Stata v. 14 (Stata Corporation, College Station, TX, USA) or GraphPad Prism version 10.0.0 for Windows, GraphPad Software, Boston, Massachusetts USA, www.graphpad.com.

Pearson’s correlation was used to investigate associations between continuous measurements.

## 3. Results

### (a) Measures of human cytomegalovirus exposure and inflammation are independently associated with risk of tuberculosis disease progression

All Ugandan participants were HCMV IgG-positive, all but five had high avidity IgG antibodies (all non-TB), and 1/49 samples from 25 TB patients and 6/242 control individuals were positive for HCMV IgM (none of whom had low HCMV IgG avidity), suggesting that very few individuals had acute HCMV infection (characterized by positive HCMV IgM) at time of sampling, but all individuals had been infected with HCMV at some point at least six months before sampling.

**Figure 2.**
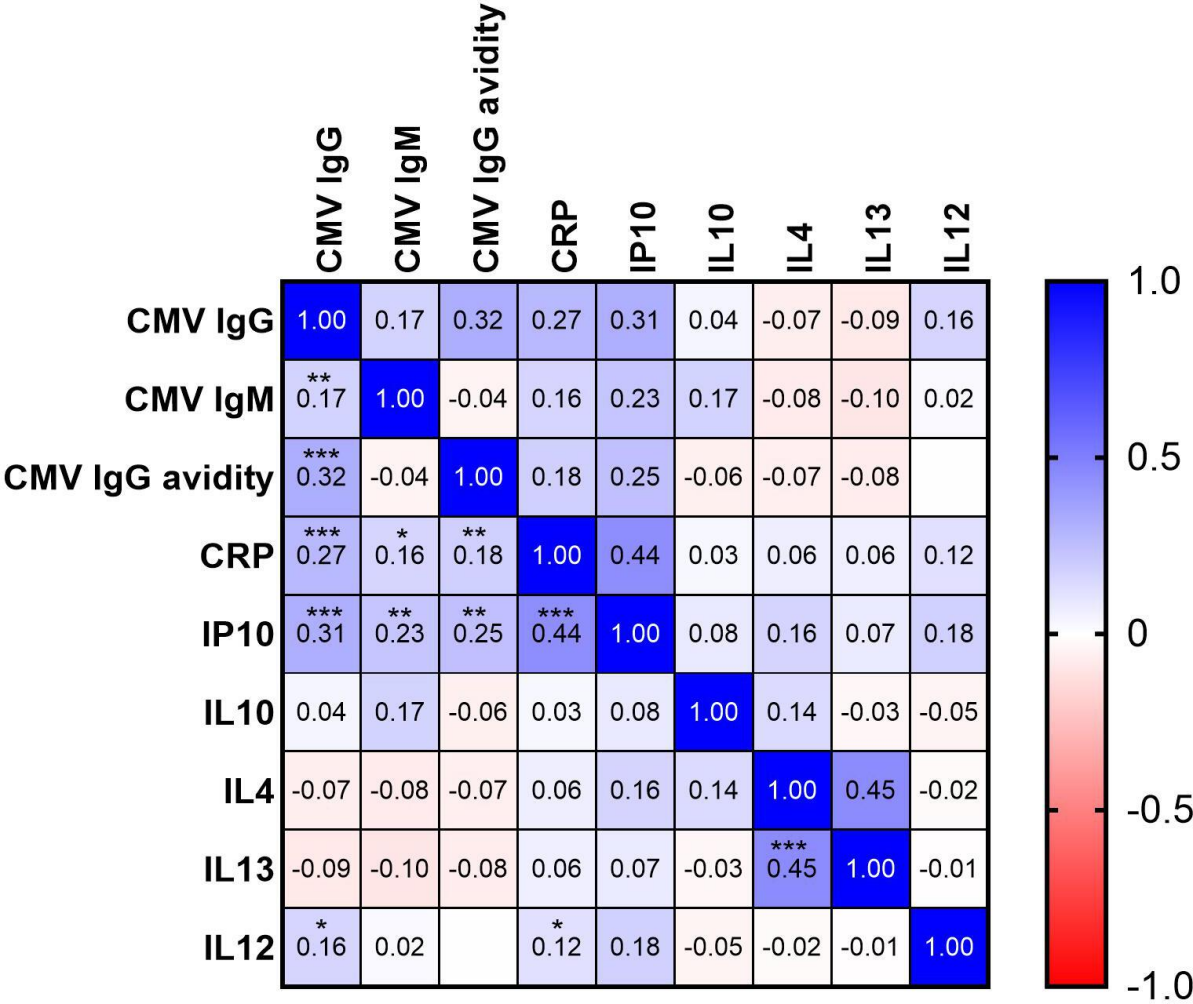
Correlation heatmap between measured analytes for the Ugandan cohort at all timepoints. * p<0.05, ** p<0.005, *** p<0.0001

We found a dose–response relationship between odds of progression to active TB disease and increased levels of HCMV IgM, HCMV IgG avidity and CRP (figure 2). We report significant positive correlations between HCMV IgG avidity and HCMV IgG, and separately, between measures of inflammation (IP10 and CRP) and both HCMV IgG avidity and HCMV IgG. To understand whether the associations seen between the odds of TB disease and HCMV IgM, HCMV IgG avidity and CRP could be being driven by HCMV IgG, we included HCMV IgG into regression models. When HCMV IgG was added into regression models, the dose–response effects seen between increased odds of progression to active TB disease with increased levels of HCMV IgM, HCMV IgG avidity and CRP levels remained (table 3), adding weight to the finding that different measures of HCMV infection and inflammation are independently associated with risk of TB disease progression in this cohort.

**Table 3.** Odds ratio of HCMV IgM, IgG avidity and CRP tertile and TB disease in the Ugandan cohort. RAI, relative avidity index.

level (range)	number of samples	OR (99% CI)	*p* value for trend	adjusted (for HCMV IgG) OR (99% CI)
HCMV IgM tertile (U)				
low (0.64−2.13)	95	1	0.0033	1
medium (2.14−3.52)	98	2.52 (0.84−7.54)		2.55 (0.80−8.11)
high (3.53−15.86)	97	3.55 (1.27−9.96)		3.31 (1.05−10.48)
HCMV IgG avidity tertile (RAI)				
low (30.72−79.34)	96	1	0.0021	1
medium (79.41−85.27)	97	2.82 (0.88−9.01)		2.72 (0.82−8.99)
high (85.33−98.04)	97	3.08 (1.25−11.82)		3.58 (1.09−11.82)
CRP tertile (mg l^−1^)				
low (0−0.180)	96	1	0.0019	1
medium (0.181−0.742)	97	1.70 (0.58−5.00)		1.66 (0.53−5.19)
high (0.745−5.25)	97	3.59 (1.20−10.74)		3.34 (1.05−10.64)

### (b) Association of human cytomegalovirus IgG levels and tuberculosis disease is maintained after adjusting for total immunoglobulin

HCMV-specific IgG-positively correlated with total IgG levels (Spearman correlation *p* < 0.001). To understand whether the association between HCMV IgG and increased odds of TB disease in the Ugandan cohort was affected by the amount of immunoglobulin circulating in that individual, total IgG levels were included in conditional logistic regression analyses (table 4). Increased odds of TB disease in medium and high tertiles of HCMV IgG were even more significant after adjusting for total IgG levels.

**Table 4.** Odds ratio of HCMV IgG and TB disease in the Ugandan cohort, adjusted for total IgG levels.

level (range)	number of samples	OR (99% CI)	*p* value for trend	adjusted (for total IgG) OR (99% CI)
HCMV IgG tertile (U)				
low (19.01−37.83)	97	1.00	0.01	1.00
medium (37.94−48.99)	97	2.63 (0.83−8.36)		2.92 (0.89−9.54)
high (49.01−103.91)	97	3.26 (0.94−11.28)		4.09 (1.07−15.65)

### (c) Measures of human cytomegalovirus infection and inflammation are not associated with increased risk of *Mycobacterium tuberculosis* infection in Gambian children

**Figure 3.**
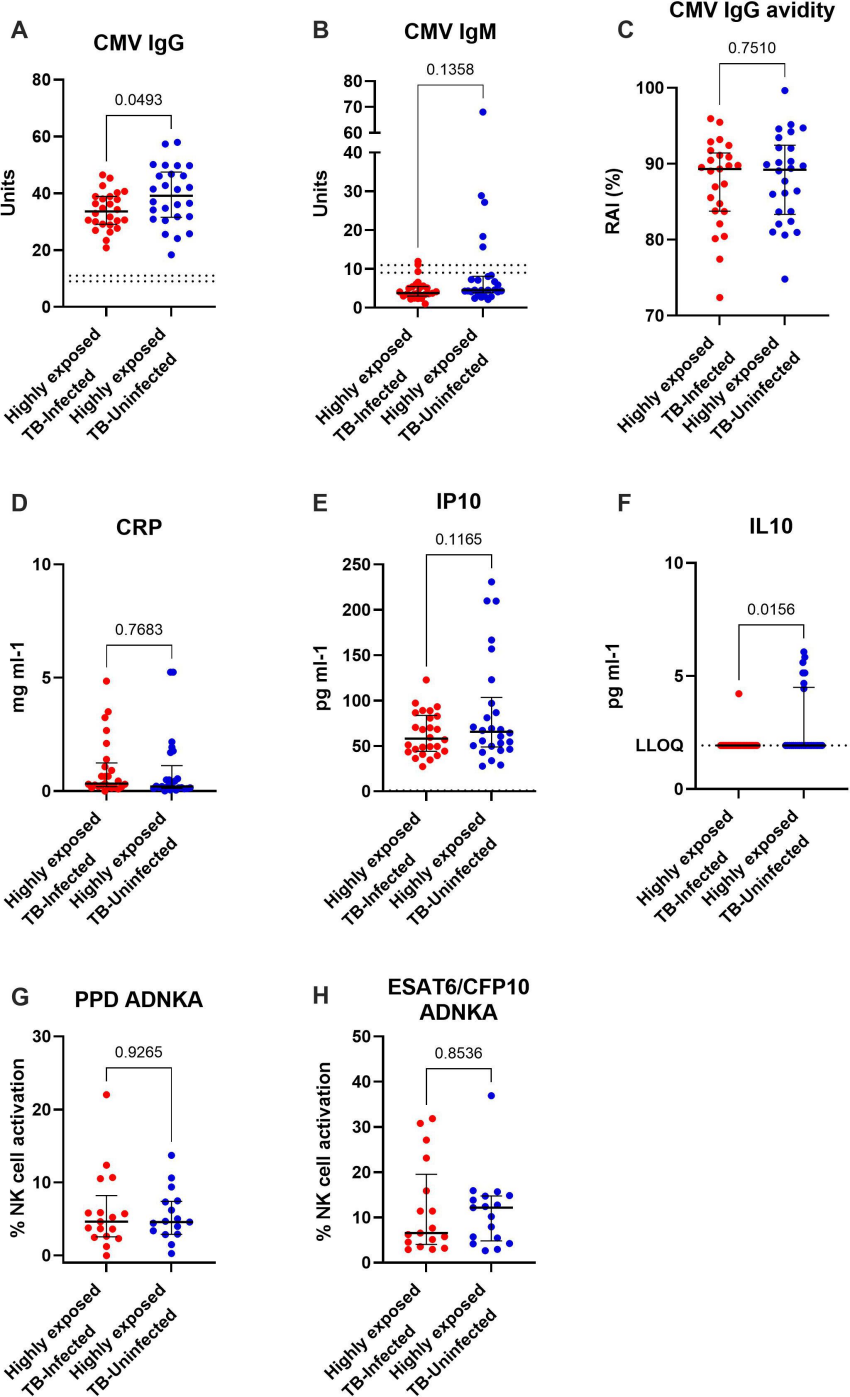
Univariate analysis comparing highly exposed–TB-infected and highly exposed–TB-uninfected children in The Gambia. Data shown only for variables for which a minimum of 15% of samples had measures above the lower limit of quantitation.

All 52 children in this Gambian cohort were HCMV IgG-seropositive. There was no statistically significant difference in age between the HEI and HEU children (*p* = 0.13). Two uninfected, and 5 *M*.*tb*-infected children were HCMV IgM-positive; however, all children had very high HCMV IgG avidity (indicative of repeated exposure), suggesting that even those seven children with positive HCMV IgM, this was not their first infection. HCMV IgM positivity did not associate with high markers of inflammation (CRP or IP10) for these 7 individuals. Figure 3 shows the differences between groups of all analytes for which data were available (no data were available for IFN-γ, IL4, IL13 and IL12 since no children had levels above the lower limit of quantitation).

Figure 3A shows that highly TB-exposed–uninfected children had significantly higher levels of HCMV IgG than matched infected children (*p* = 0.05), which is opposite to the direction seen among the Ugandan cohort. Only 4/52 Gambian children had measurable levels of HCMV vIL10 and so they were not included in analyses. IL-10 levels were significantly elevated among highly TB-exposed–uninfected children compared with highly TB-exposed–infected children (*p* = 0.02, figure 2F); however, only 8/52 children had IL-10 levels above the lower limit of quantitation.

**Figure 4.**
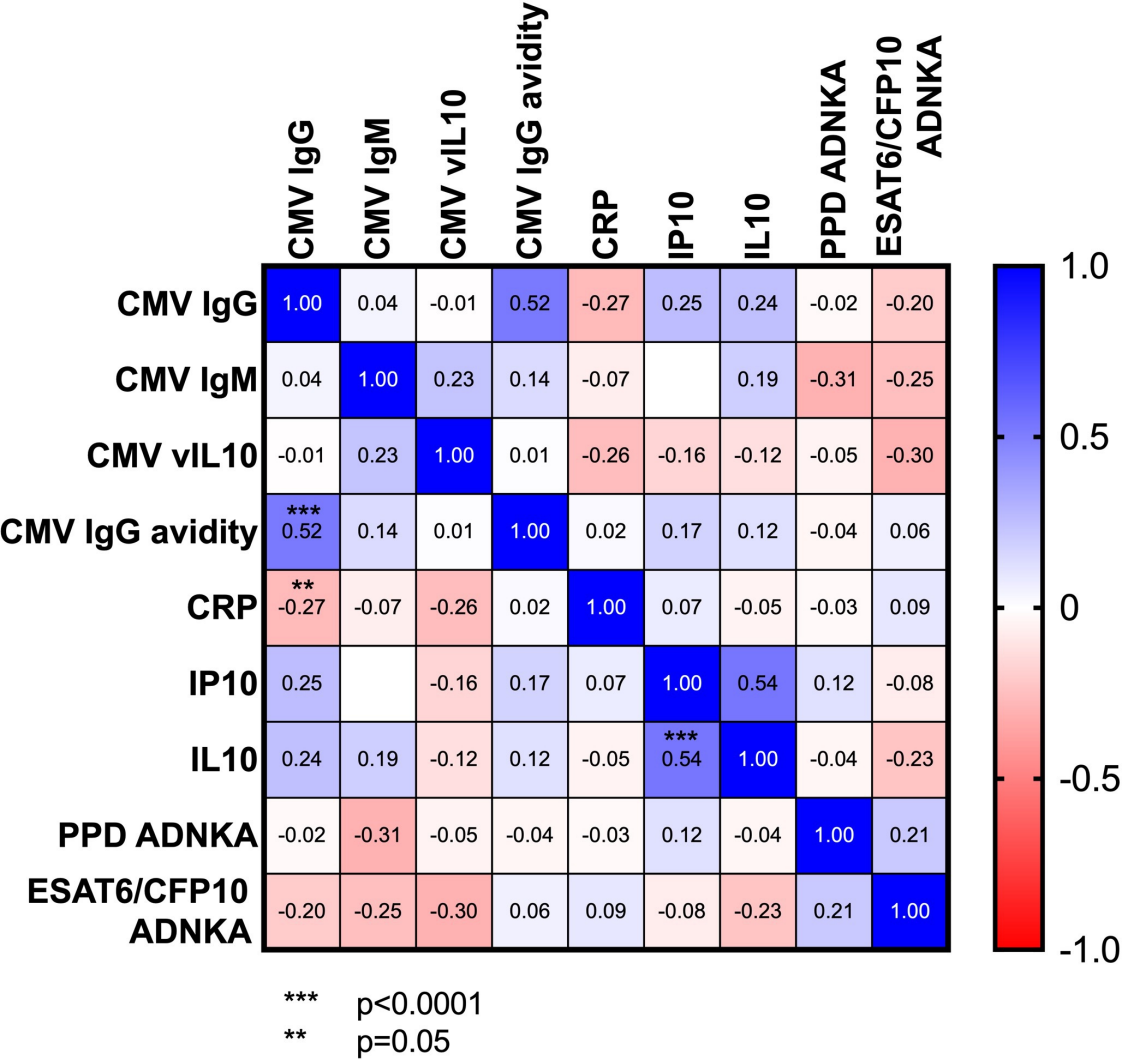
Correlation heatmap showing variables for which a minimum of 15% of samples from Gambian highly exposed–TB-infected and highly exposed–TB-uninfected children reported measures above the lower limit of quantitation.

The ability of mycobacteria-specific antibodies (purified protein derivative (PPD) or ESAT6/CFP fusion protein) to activate NK cells did not differ between Gambian children who were either infected, or not, with *M.tb* (figure 3G,H). Additionally, there was no association between the ability of mycobacteria-specific antibodies to induce NK-cell activation and any measures of HCMV, or inflammation (figure 4).

## 4. Discussion

Using a nested case–control study in Ugandan individuals, we show that alternative measures of HCMV infection (IgM and IgG avidity) are associated in a dose-dependent manner with the risk of developing TB disease in samples taken up to 10 years before TB diagnosis. This is consistent with our prior data, which also found a dose–response relationship between increased HCMV IgG and risk of TB disease [9]. CRP—a measure of systemic inflammation—was also independently associated with increased risk of developing TB disease. The distinct contributions of HCMV and elevated CRP are difficult to disentangle as HCMV is often associated with increased inflammation [27]. There is however considerable heterogeneity of CRP levels among HCMV-infected individuals. Indeed, in a large US population-based study, HCMV was associated with a significantly increased risk for all-cause mortality; however, HCMV-seropositive subjects who also had highCRP levels were at substantially higher risk for both for all-cause and cardiovascular disease-related mortality than subjects with low CRP levels [27].

In the Ugandan nested case–control cohort, when adjusting for HCMV IgG levels, we report 3.55 and 3.62 times increased risks of TB disease in individuals with the highest tertile of HCMV IgM and IgG avidity (respectively). Additionally, we see a 3.59 times increased risk among individuals with the highest tertile of CRP. Given that all individuals were HCMV-seropositive (as measured by IgG), we are unable to tease apart the effect of HCMV infection and high inflammation from other causes. The utility of HCMV IgG levels as a composite measure of HCMV infection, reinfection and reactivation events is debated. As far as we know, there is no literature directly comparing measures of presence of the virus and IgG. The finding here that the association between risk of TB disease and HCMV IgG remains after adjusting for total IgG levels provides evidence that HCMV-specific IgG, and not an HCMV-B-cell activation, leading to increased production of IgG more generally, is associated with risk of progression to TB disease.

As was reported previously by the group in the same Ugandan cohort, the finding of association between HCMV IgG and TB disease was not detected for other herpes viruses, Epstein Barr virus or herpes simplex virus [9]. There are a variety of possible mechanisms by which HCMV infection may exacerbate *M.tb* infection and lead to increased risk of TB disease (reviewed in [22]). HCMV encodes viral proteins that may interfere with protective immune responses. UL111A, a homologue to the immunosuppressive cytokine IL-10, is perhaps most important for TB [28]. As a functional, but not structural homologue, we hypothesize that hcmvIL-10 could exert its role to suppress macrophage and dendritic cell functions, which is essential to the phagocytosis of M.tb bacilli and initiation of protective immune responses [29] , but meanwhile be undetectable by diagnostics specific to human IL-10 detection.

Despite the careful exposure-matched study design of children included in the Gambian cohort, we do not find an association between measures of HCMV infection and cumulative exposure and risk of infection with *M.tb*. These data corroborate findings from a larger South African cohort of BCG-vaccinated infants that also did not find evidence of an association between HCMV and infection with *M.tb* [12]. Immunologically, it might be hypothesized that HCMV infection, reinfection and reactivation (here hypothesized to be associated with comparatively elevated levels of HCMV IgG) might be associated with differential ability to mount a response to *M.tb* antigens, which form the basis of the diagnostics for identification of *M.tb* infection. When monozygotic twins were investigated for non-heritable influences of immune variation, HCMV infection was found to impact many immune features [17]. HCMV infection has such an effect on the T-cell population that in addition to a general increase in effector memory cells [30], over 10% of circulating T cells have been found to be directed towards HCMV in infected individuals [31]. We are unable to disentangle a possible anergic TST or IGRA response that might impede diagnosis of *M.tb* infection in this cohort. If found, such an interaction would be a serious drawback to current diagnostic methods. With the data available here, it is difficult to adjust for all potential confounders and so we cannot attribute a causal relationship between HCMV, inflammation and *M.tb* responses in our cohorts. We do however contribute to the body of evidence and biological plausibility that the immunological interaction between HMCV infection and *M.tb* exposure should be further explored with experimental models to test for causal relationships and validation in larger and additional prospective cohorts.

Some of the limitations of this study include that within the Ugandan cohort, control samples were matched to case samples on age, sex and HIV status when the sample was taken as opposed to matching at point of diagnosis, which may introduce a potential source of bias. Reliance upon passive TB case detection in the GPC meant that control individuals included in this study were not investigated for *M.tb* infection. The grouping into tertiles of HCMV-specific IgM and IgG avidity, and CRP, is based upon the ranges seen in this population and the exact ranges of levels may not be found using alternative commercial kits, or indeed be generalizable to other populations. Because the timing of sampling of the Ugandan cohort was directed by other research questions, there is a risk that the TB disease outcome measure may encapsulate both risk of infection and risk of progressing from infection to disease.

The careful exposure-matched study design is a strength of the Gambian child cohort. However, the parent study was designed to detect a difference in mycobacterial luminescence in a whole blood assay with 80% power, and there was a limited number of pairs of children from whom sufficient sample was available for experimental analysis here. Therefore, the small sample size means that this exploratory analysis is likely underpowered. In this study, we used TST positivity to determine TB infection status. In-house IGRA status was available at baseline for the highly TB-exposed–infected children but was not available for the highly TB-exposed–uninfected children. An overarching limitation of this study, and of the TB field in general, is the poor tools we have for detecting *M.tb* infection [32].

In summary, this work extends the positive association between HCMV infection and the magnitude of exposure with risk of progression to TB disease in a Ugandan cohort. The data presented here show the importance of evaluating the potential impact of HCMV vaccines against TB disease and larger prospective studies accompanied by functional *in vitro* experiments are required to establish clinical relevance and whether the relationship is more than association.

## Data Availability

Data are available at [33].
